# A Manual of Instructions

**Published:** 1903-04

**Authors:** 


A Manual of Instructions in the Principles of Prompt Aid to the Injured, including a Chapter on Hygiene and the Drill Regulations for the Hospital Corps, U. S. A., Designed for Military and Civil Use by Alva H. Doty, M. D., Health Officer of the Port of New York, etc. Fourth edition, revised and enlarged; 302 pages. Price $1.00. New York, D. Appleton & Co., 1902.

The general plan of the arrangement of subject matter in the present edition is similar to that adopted in former editions. Such changes in the text as would be necessary to cause conformity to our present knowledge have been made. The chapter on Disinfection has been rewritten. The Hospital Corps Drill Regulations now used by the U. S. Army have been introduced. The work is a valuable one. T. J. B.

